# Pain and Cellular Migration Induced by *Bothrops jararaca* Venom in Mice Selected for an Acute Inflammatory Response: Involvement of Mast Cells

**DOI:** 10.3389/fimmu.2021.779473

**Published:** 2022-02-04

**Authors:** Fernanda V. Kondo, Wafa H. K. Cabrera, Orlando G. Ribeiro, Marcelo De Franco, José Ricardo Jensen, Gisele Picolo, Morena B. Sant’Anna, Monica Spadafora-Ferreira, Andrea Borrego, Olga M. Ibañez, Nancy Starobinas

**Affiliations:** ^1^Laboratory Immunogenetics, Butantan Institute, São Paulo, Brazil; ^2^Diagnostic Section, Pasteur Institute, São Paulo, Brazil; ^3^Laboratory of Pain and Signaling, Butantan Institute, São Paulo, Brazil

**Keywords:** snake venom, mast cells, genetically selected mice, hyperalgesia, acute inflammation

## Abstract

*Bothrops jararaca* venom (BjV) can induce mast cell degranulation. In order to investigate the role of mast cells and the interference of the host genetic background in the inflammation induced by BjV, we have used mouse strains selected for maximal (AIRmax) or minimal (AIRmin) acute inflammatory response (AIR). Mice were pretreated with an inhibitor of mast cell degranulation, cromolyn (CROM), and injected in footpads or intraperitoneally (i.p.) with BjV. Pain was measured with von Frey hairs, cell migration in the peritoneum by flow cytometry, and reactive oxygen species (ROS) production by chemiluminescence assays. The nociceptive response to BjV was higher in AIRmax than AIRmin mice; however, this difference was abolished by pretreatment with CROM. BjV induced peritoneal neutrophil (CD11b^+^ GR-1^+^) infiltration and ROS secretion in AIRmax mice only, which were partially inhibited by CROM. Our findings evidence a role for mast cells in pain, neutrophil migration, and ROS production triggered by BjV in AIRmax mice that are more susceptible to the action of BjV.

## Introduction

The large majority of ophidic accidents in Brazil are caused by *Bothrops jararaca* (Bj) snakes (1). Pathogenesis of *Bothrops* envenomation involves the combined action of venom metalloproteinases, phospholipases, and serine proteinases, as well as the release of endogenous mediators originated either from plasma or from inflammatory cells (2–4).

The inflammatory response resulting from tissue damage and the direct action of toxins is characterized by a local increase in blood flow, exudation of plasma proteins, and migration and activation of leukocytes from the vessels to the site of injury. Since many of these inflammatory effects are due to the release of chemical mediators from injured tissue, mast cells have been considered important in the onset and/or amplification of acute inflammatory responses (5–7).

Metalloproteinases and phospholipases from *Bothrops jararaca* venom (BjV) can induce mast cells to release potent biologically active mediators present in their granules (histamines, proteoglycans, and proteases), promoting inflammation with an increase in vascular permeability, the expression and/or activation of adhesion molecules with a rapid local recruitment of neutrophils, pain, clotting and fibrinolysis systems abnormalities, and shock (4, 8–14).

In order to investigate the role of mast cells in BjV-triggered inflammatory reaction and the interference of host genetic background in this effect, we have used mouse lines phenotypically selected for maximum (AIRmax) or minimum (AIRmin) acute inflammatory response (AIR) (15, 16). At the end of the bidirectional selective process for the production of AIRmax and AIRmin lines, the gene alleles endowed with opposite effects in the regulation of the maximum and minimum acute inflammatory responses were considered to be accumulated in AIRmax and AIRmin mice, respectively (16). Therefore, these mouse lines are normal and represent the individuals who are situated at the extremes of high or low response that are found in heterogeneous populations.

As a result of their divergent inflammatory response, we have demonstrated that AIRmax and AIRmin mice differ in their susceptibility to bacterial infections (17), tumorigenesis (18, 19), and autoimmune diseases (20, 21). With regard to systemic and local responses induced by BjV, both lines are equally susceptible to venom-induced lethality, showing similar LD_50_. However, edema, leukocyte influx, prostaglandin E_2_ (PGE_2_) secretion, hydrogen peroxide (H_2_O_2_) production, Chemokine (C-X-C motif) ligand 2 (CXCL2)/macrophage inflammatory protein (MIP)-2 expression, and interleukin (IL)-1β and IL-6 release are more intense in AIRmax than those in AIRmin mice (22, 23).

Based on previous literature showing the role of mast cells in response to venom or its components, our aim in this study was to evaluate the participation of these cells in BjV-induced inflammatory parameters such as hyperalgesia, cellular migration, and reactive oxygen species (ROS) production in mice selected for maximum and minimum inflammatory response.

## Materials and Methods

### Mice

AIRmax and AIRmin mice weighing 25 g, male or female, were obtained from the animal facilities of the Immunogenetics Laboratory. The animals were maintained under a standard diet and water *ad libitum*, with controlled temperature (21°C ± 2°C), humidity (50% ± 10% RH), and light cycle (12/12-h light/dark), in a soundproof room.

These mice were selected for acute inflammatory response using the subcutaneous injection of polyacrylamide beads (Biogel P-100) as an inflammatory stimulus and the number of local inflammatory cells and exudated protein concentration as the selection phenotypes as previously described (15). After about 25 generations of selective breeding, AIRmax and AIRmin lines showed 25-fold and 2.5-fold differences in the number of infiltrating leukocytes and protein concentrations in the local inflammatory exudate, respectively, between them (16).

Behavioral tests were carried out between 9:00 a.m. and 5:00 p.m. All procedures were approved by the Ethics Committee on Animal Use of the Butantan Institute (CEUAIB protocol number 1213/14 and 9966041019) and carried out in accordance with the ethical use of conscious animals in pain research published by the International Association for the Study of Pain (24) and the guideline of the National Institutes of Health Guide for the Care and Use of Laboratory Animals (8th Edition 2011).

### Venom and Pharmacological Treatment

Lyophilized crude BjV was supplied by the Herpetology Laboratory of the Butantan Institute. Cromolyn [sodium cromoglycate (CROM)] and compound 48/80 (a compound known to degranulate mast cells) were purchased from Sigma–Aldrich (Brazil). Mice received intraperitoneal (i.p.) CROM (100 mg/kg) injections dissolved in phosphate buffered saline (PBS) for 3 consecutive days before BjV or compound 48/80 (C48/80 1 mg/kg, i.p.) injection on the fourth day. Control mice received PBS i.p. for 3 consecutive days before BjV or compound 48/80 injection.

**Figure 1:**
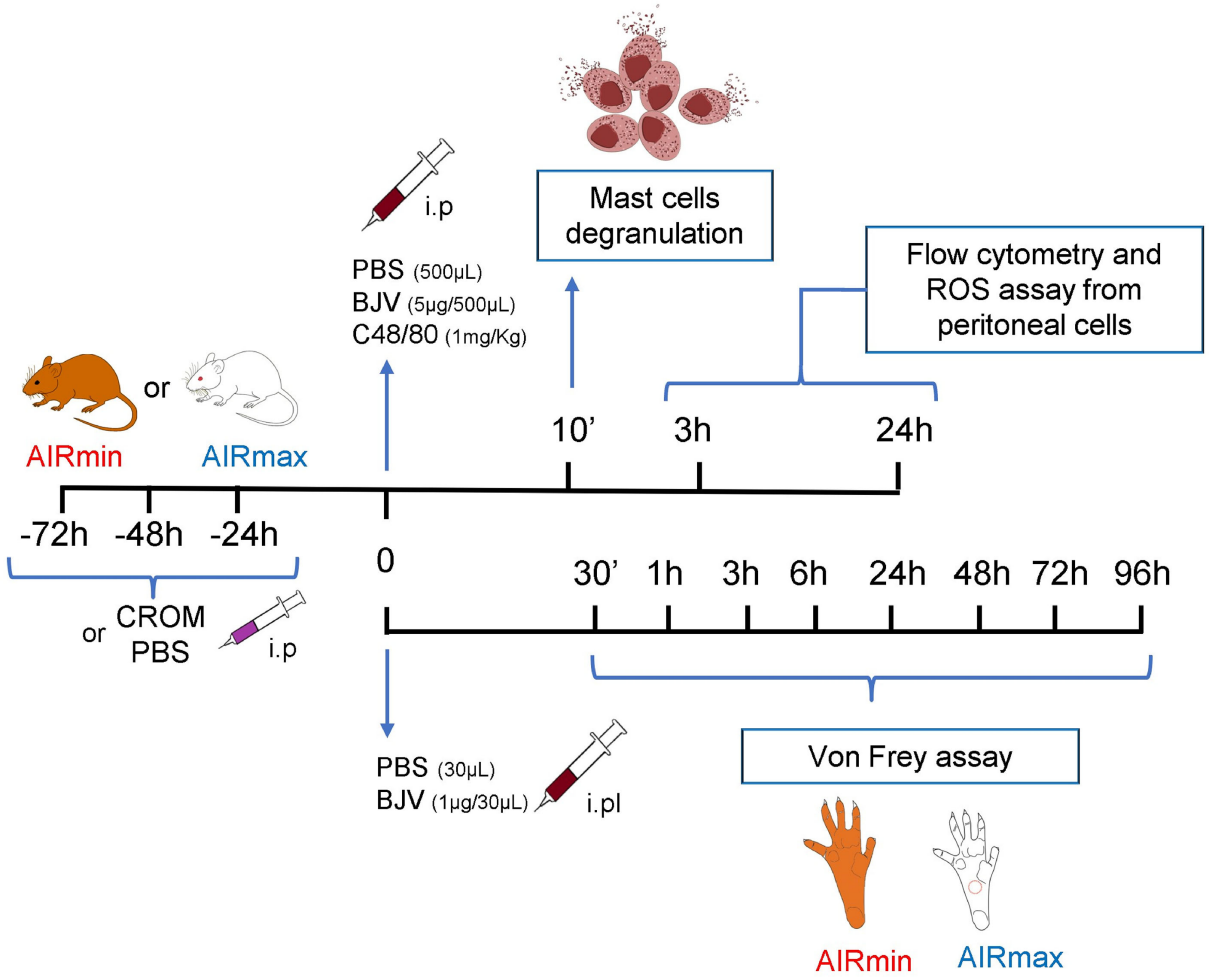
Experimental protocol scheme.

The experimental strategy, where the pharmacological groups and biological fluids/tissues analyzed, is presented (**Figure 1**).

### Mast Cell Degranulation

Mice pretreated with CROM (100 mg/kg) for 3 days received PBS, compound 48/80 (1.0 mg/kg), or BjV (5.0 µg). After 10 min, mice were euthanized in a CO_2_ chamber, the abdomen was opened and the mesentery was carefully removed, fixed in a 4% paraformaldehyde solution for 24 h, washed, stained with Toluidine blue, and mounted on a glass slide. Degranulation was expressed as the proportion (%) of mast cell-extruded granules relative to total mast cells present in the tissue. At least 100 cells were counted in microscopy (×250 magnification). Control mice received PBS i.p., n = 5–8 animals/group.

### Hyperalgesia Assessment

Male mice were injected with BjV (1.0 µg/30 µl sterile apyrogenic saline, i.pl.) into the right hind paw. Control animals were injected with saline only. Animals were placed on an elevated wire grid and habituated to the experimental environment for a minimum of 20–30 min. Pain mechanical threshold was accessed at 0.5, 1, 3, 6, 24, 48, 72, and 96 h after injection using von Frey hairs, as previously described (25, 26). Briefly, the paw withdrawal threshold was taken as the lowest force that evoked a brisk withdrawal response to one of five repetitive stimuli. A logarithmic series of 9 calibrated monofilaments (von Frey hairs) was applied to the right hind paws to determine the stimulus intensity threshold stiffness required to elicit a paw withdrawal response. The log10 stiffness of the hairs ranged from 0.903 (8 mg) to 3.146 (1,400 mg).

### Flow Cytometry Analysis

To analyze the inflammatory reaction in peritoneal cavity, mice were injected i.p. with 5 µg BjV in 0.5 ml PBS. After 3 or 24 h, the animals were euthanized in a CO_2_ chamber and the peritoneal cavities were washed with 5 ml PBS. Control animals were injected with 0.5 ml PBS. Total cells were counted in Malassez hemocytometric chambers. Peritoneal cells (1 × 10^6^) were incubated with fluorochrome-conjugated monoclonal antibodies specific for FcϵRI, cKIT, CD11b, F4/80, Gr-1, and Major Histocompatibility Complex (MHC)-II (BD Biosciences and BioLegend) labeled with Fluorescein isothiocyanate phycoerythrin (FITC), phycoerythrin (PE), Pacific blue, Allophycocyanin (APC), or AmCyan and acquired in a FACSCanto II (BD Biosciences) flow cytometer. FITC rat IgG2a (BD Pharmingen) and PE rat IgGk (BD Pharmingen) were used as control isotypes. The results were analyzed with FlowJo software, version 10.1 (Becton-Dickinson), mast cells (FcϵRI^+^ cKIT^+^), neutrophils (CD11b^+^ GR-1^+^), and macrophages (CD11b^+^ GR-1^low^ F4/80^+^ MHC-II^+^). The experiment was performed 3 times with 3 animals per group. The results were calculated by multiplying the frequency of the population (%) obtained from flow cytometry data by the total number of peritoneal live cells enumerated in each sample.

### Reactive Oxygen Species Production Detection

Activation of peritoneal cells was evaluated by ROS production measured by a luminol-based chemiluminescent probe (27). Briefly, cells (1 × 10^5^), luminol (100 μM, Sigma-Aldrich), phorbol myristate acetate (PMA; 90 nM, Sigma-Aldrich), and PBS were added to white 96-well plates (Costar), and readings (during 3,600 s for 130 cycles) were carried out in an EG&G Berthold LB96V microplate luminometer at room temperature. Results were expressed as the area under the curve of relative light units (RLUs) using Micro Win Software (Mikrotek Laborsystems). The results are expressed as mean ± SEM.

### Statistical Analysis

Statistical analysis was performed by analysis of variance (ANOVA), followed by Tukey multiple comparison tests or unpaired t test with Welch’s correction (GraphPad Software, San Diego, CA, USA; EUA v. 5.01). Results were considered statistically significant when *p < 0.05, **p < 0.01, ***p < 0.001.

## Results

**Figure 2:**
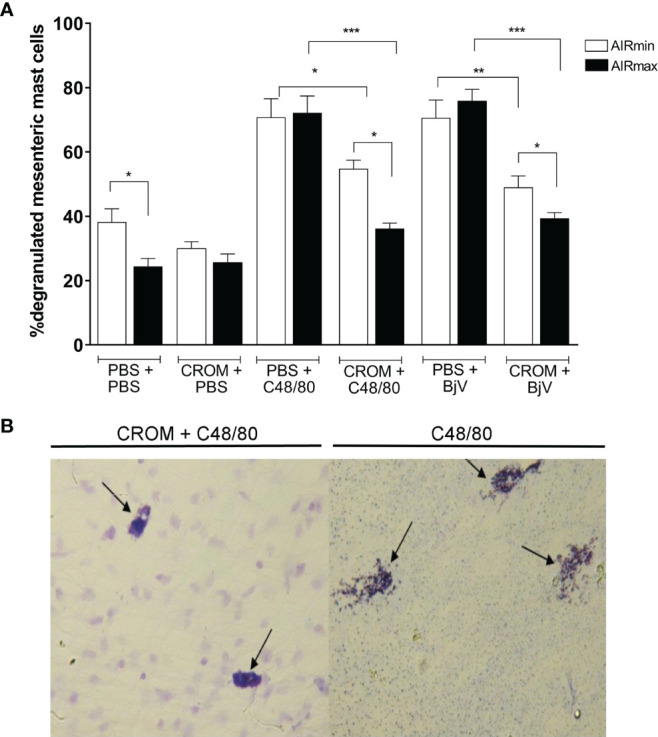
Effect of cromolyn (CROM) on mast cell degranulation induced by phosphate buffered saline (PBS), C48/80, or *Bothrops jararaca* venom (BjV) in AIRmax and AIRmin mice. Percentage of degranulation of mesenteric mast cell **(A)**. Illustration of CROM effect in AIRmax mice. **(B)** Mice pretreated with CROM (100 mg/kg) for 3 days received PBS, compound 48/80 (1.0 mg/kg), or BjV (5.0 µg). Mesenteric tissue was removed after 10 min, degranulated mast cells were measured by count in microscopy. Toluidine blue staining. Control mice received PBS i.p., n = 5–8 animal/group. Unpaired t test with Welch’s correction *p < 0.05, **p < 0.01, ***p < 0.001 indicate a significant difference.

To certify that CROM inhibits mast cell degranulation in our model, we analyzed the mesenteric tissue of AIRmax and AIRmin mice pretreated with CROM and injected with PBS, BjV, or C48/80, a compound known to degranulate mast cells. Despite the difference observed between PBS-injected AIRmax and AIRmin, the induction of mast cell degranulation by both C48/80 and BjV was similar in both strains. However, CROM inhibited 49.8% and 22.6% of C48/80-induced mast cell degranulation in AIRmax and AIRmin mice, respectively. Similarly, pretreatment with CROM inhibited 48% and 30.6% of BjV-induced degranulation in AIRmax and AIRmin, respectively (**Figure 2A**).

A visual illustration of CROM effect can be found in **Figure 2B**, where we show degranulated mesenteric mast cells collected from AIRmax mice after C48/80 injection. We observed a similar aspect in cells collected from mice injected with BjV, and this was not different from what we observed in AIRmin mice (image not shown).

**Figure 3:**
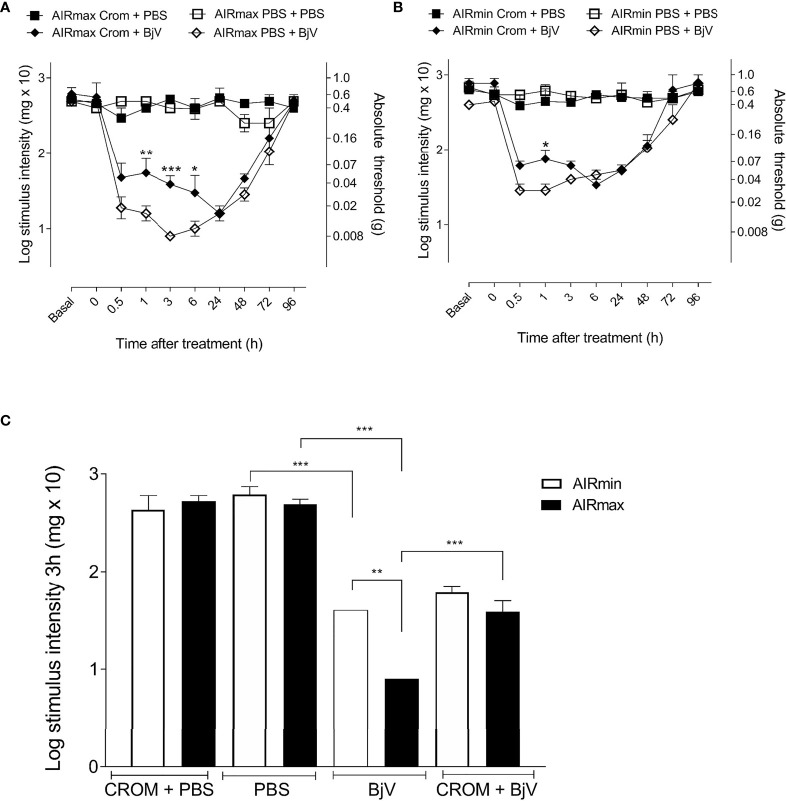
Evaluation of cromolyn (CROM) treatment in hyperalgesia and mast cell degranulation. AIRmax **(A)** and AIRmin **(B)** mice were pretreated with CROM (100 mg/kg) and inoculated with *Bothrops jararaca* venom (BjV) (1 µg) in the hind paw, and pain was tested with von Frey hairs. **(C)** Hyperalgesic response of AIRmax and AIRmin mice at 3 h post-BjV injection. Each bar represents the mean ± SEM of 4 animals. Panels **(A**, **B)** *p < 0.05, **p < 0.01, ***p < 0.001 indicate a significant difference between CROM+BjV and PBS+BjV. Panel **(C)** **p < 0.01, ***p < 0.001 indicate differences between BjV and PBS, CROM+BjV and BjV, and between AIRmax and AIRmin mice. The different groups are indicated in the figure. One-way ANOVA with Tukey’s posttests.

The injection of BjV (1 µg/hind paw) induced significant hyperalgesia in both lines (**Figures 3A, B**); however, it was more intense in AIRmax compared to AIRmin mice (**Figure 3C**). Hyperalgesia peaked at 3 h in AIRmax and between 30 min and 1 h in AIRmin mice. Pretreatment with CROM partially inhibited BjV-induced hyperalgesia for up to 6 h in AIRmax and 1 h in AIRmin, with similar residual hyperalgesia in both lines (**Figures 3A, B**). Pain levels were unchanged in control groups at all time points.

The difference in nociceptive response to BjV between AIRmax and AIRmin mice was abolished by pretreatment with CROM, represented at the third hour (**Figure 3C**). These results suggest a significant contribution of mast cells in the differences in pain threshold observed between AIRmin and AIRmax mice after injection of BjV.

**Figure 4:**
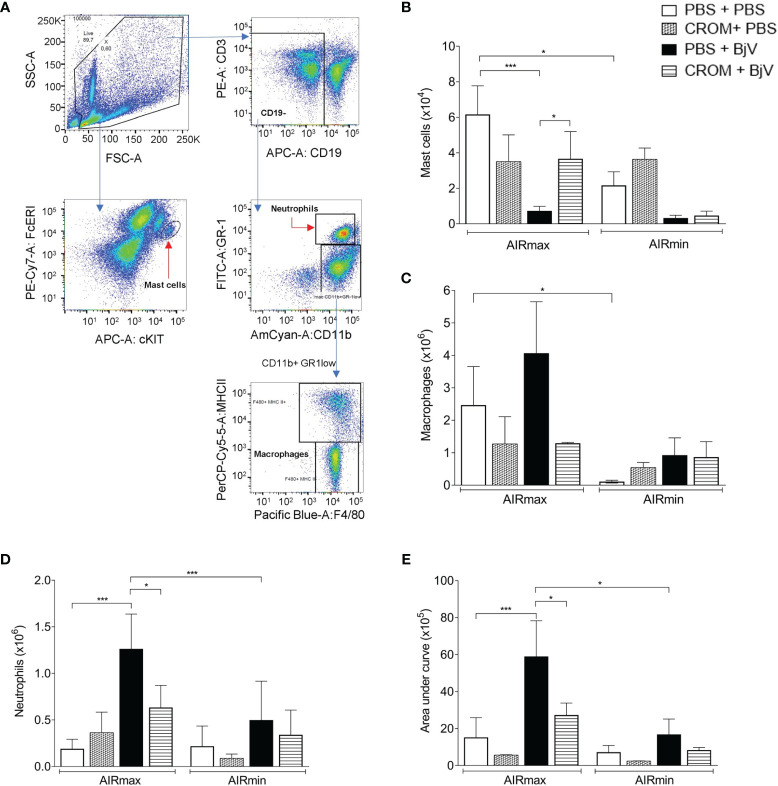
Effect of cromolyn (CROM) treatment on peritoneal mast cells, macrophages, neutrophils, and reactive oxygen species (ROS) production induced by *Bothrops jararaca* venom (BjV). AIRmax and AIRmin mice were pretreated with CROM (100 mg/kg) or phosphate buffered saline (PBS) and then inoculated with BjV (5 µg i.p.) or PBS. Flow cytometry gated strategy analysis **(A)**. Mast cells (FcϵRI^+^ cKIT^+^) 3 h after inoculation **(B)**, macrophages (CD11b^+^ GR-1^low^ F4/80^+^ MHC-II^+^) **(C)**, and neutrophils (CD11b^+^ GR-1^+^) **(D)** obtained 24 h after BjV injection. ROS production **(E)** was evaluated by chemiluminescence during 1 h of phorbol myristate acetate (PMA) stimulation. Data are expressed as mean ± SEM of 3–6 animals/group. *p < 0.05, ***p < 0.001 indicate a significant difference. One-way ANOVA with Tukey’s posttests.

Flow cytometry was used to analyze the cellular profile infiltrated into the peritoneal cavity after BjV inoculation. The gate strategy for the analysis is represented in **Figure 4A**. The numbers of peritoneal mast cells (FcϵRI^+^ cKIT^+^) in PBS-treated mice were higher in AIRmax than in AIRmin mice and decreased after BjV injection. Degranulated cells could not be detected by flow cytometry. On the other hand, pretreatment with CROM increased mast cell numbers in BjV-injected AIRmax but not in AIRmin mice likely due to the inhibition of their degranulation (**Figure 4B**). Pretreatment with CROM did not alter the number of mast cells in the peritoneal cavity of PBS controls.

The peritoneal macrophage population (CD11b^+^ GR-1^low^ F4/80^+^ MHC-II^+^) in control mice was higher in AIRmax mice compared to AIRmin mice. This population was not significantly altered by any treatment in both mouse lines (**Figure 4C**). On the other hand, infiltrated neutrophil population (CD11b^+^ GR-1^+^) was increased in AIRmax mice after BjV inoculation but not in AIRmin and was partially inhibited by CROM (**Figure 4D**).

Since macrophages and neutrophil’s function can be modulated by mast cells, we quantified PMA-stimulated ROS production as a method to evaluate the effect of BjV on peritoneal cell activation. AIRmax cells produced higher levels of ROS than AIRmin cells 24 h after i.p. venom injection. CROM pretreatment inhibited ROS production in AIRmax but not in AIRmin, suggesting the involvement of mast cells in BjV-induced peritoneal cell activation (**Figure 4E**).

## Discussion

In this work, we have investigated the role of mast cells in some important parameters of the inflammatory response triggered by BjV in mice genetically selected for inflammation, AIRmax and AIRmin.

Mast cells are considered the primary storage site of histamine in tissues from mammalians. Histamine is stored, together with serotonin, in secretory granules, formed by a highly charged matrix of heparin and protein (28). These cells can be degranulated by a range of agents, including direct action of phospholipases hydrolyzing membrane phospholipids (29), complement fragment 3a (C3a) that induces degranulation stimulated by aggregated IgG, calcium-dependent exocytosis process, and also diverse agents that induce degranulation through Toll-like receptor 2 (TLR-2) activation (30, 31).

Pain is an important signal present during an inflammatory response, and BjV injection induced a significant hyperalgesia in both lines, being more intense in AIRmax mice. Our previous studies have shown the presence of high levels of PGE_2_ in the peritoneal cavity of AIRmax mice after BjV i.p. injection (22). Venom components such as phospholipase A_2_ (PLA_2_) could lead to prostaglandin synthesis (32, 33), and this higher level of PGE_2_ associated with mast cell degranulation could partially explain the more intense BjV-induced pain observed in AIRmax in comparison to AIRmin mice. Accordingly, the treatment with CROM partially inhibited BjV-induced hyperalgesia, indicating the contribution of mast cells to the nociceptive response.

The inhibition of BjV-induced pain by CROM could be compared to results where CROM inhibited mast cell degranulation and pain induced by Freund’s complete adjuvant in mice (34). It was already reported, in rats, that the hyperalgesia triggered by BjV is inhibited by meclizine and methysergide, antagonists of histamine (H1) and 5-hydroxytriptamine (5-HT1, 5-HT2, and 5-HT7) receptors, respectively (4). The same authors showed the involvement of mast cells, since CROM inhibited BjV-induced thermal hyperalgesia. The mechanism responsible for BjV-induced mast cell activation was associated with the metalloproteinase component of the venom. Previous treatment with metal chelator abolished the venom effect on mast cells (4). Batroxase, a P-I metalloproteinase isolated from *Bothrops atrox* venom, induced the edematogenic and hyperalgesic responses involving mast cell degranulation and histamine and leukotrienes as mediators (35). Bernardes et al. (8) analyzed BpirMP, a metalloproteinase isolated from the venom of the *Bothrops pirajaí* snake, and observed induction of paw edemas and increased nociceptive threshold in rats, which were inhibited by pretreatment with CROM. Our results confirm and extend these observations, since we have used a different animal model and added the interference of genetic components on the evaluated phenotype.

The higher BjV-induced infiltration of neutrophils in AIRmax mice is in accordance with the phenotype used in the selection of these animals. Our group demonstrated previously that in response to an inflammatory stimulus, bone marrow neutrophil production was more intense, concentration of chemotactic factors in inflammatory exudates was higher, and neutrophil resistance to apoptosis was increased in AIRmax mice compared to AIRmin (36). Other authors have described that venom injection induces chemotaxis (37), which can be regulated by mediators released by mast cells, promoting effector functions in Polymorphonuclear leukocytes (PMN) cells (10, 38).

Landucci et al. (39) demonstrated that PLA_2_ enzymes were involved in mast cell activation caused by *Bothrops jararacussu* venom. PLA_2_ isolated from the venom of *Lachesis muta muta* including LmTX-I induces microvascular leakage mediated by mast cell activation when administered *via* intraperitoneal or intradermal routes (40). The products secreted by mast cells are thus important in the worsening of the injury, and they can be an important therapeutic target (41). On the other hand, some authors have shown that products secreted during the mast cell degranulation can counteract the toxicity of the venoms, especially proteinases, which cleave some of these toxins (42).

Mast cell activation was recently shown to be involved in the early inflammatory response of mesenteric microvasculature to lipopolysaccharide (LPS) inoculation in mice. Treatment with the non-specific mast cell stabilizer CROM prevented mast cell activation toward a pro-inflammatory phenotype, significantly reducing the number of migrated neutrophils (11). Our results suggest that during venom stimulation of AIRmax mice, something similar is occurring, with mast cells contributing to neutrophil migration, since this process is modulated by CROM treatment. We did not observe these effects in AIRmin mice at the time points and doses measured. These mice presented a mild inflammatory response after BjV inoculation in comparison to AIRmax.

In conclusion, our study suggests that mast cells play a role in pain, neutrophil migration, and ROS production triggered by BjV. AIRmax mice are more susceptible than AIRmin mice to BjV-induced inflammatory reactions, which are partially inhibited by pretreatment with CROM, a mast cell stabilizer. The use of these genetically selected mouse strains endowed with extreme divergent inflammatory responses could contribute to a better understanding of the effect that these venoms have in our organism. Clinical reports indicate that predictive factors of worsening of snake poisoning are very variable and difficult to prove; however, it is considered that variations in the genetic constitution of the victim and the venom composition can interfere in the severity and outcome of poisoning (43).

These results point to a possible beneficial effect of drugs that modulate the activation of mast cells in BjV envenomation.

## Conflict of Interest

The authors declare that the research was conducted in the absence of any commercial or financial relationships that could be construed as a potential conflict of interest.

## Publisher’s Note

All claims expressed in this article are solely those of the authors and do not necessarily represent those of their affiliated organizations, or those of the publisher, the editors and the reviewers. Any product that may be evaluated in this article, or claim that may be made by its manufacturer, is not guaranteed or endorsed by the publisher.

## Data Availability Statement

The raw data supporting the conclusions of this article will be made available by the authors without undue reservation.

## Ethics Statement

The animal study was reviewed and approved by the Ethics Committee on Animal Use of the Butantan Institute.

## Author Contributions

FK and NS conceived and designed the experiments. FK, MS’A, WC, and AB performed the experiments. NS, FK, MS-F, MS’A, and GP analyzed the data. NS, OR, MF, and OI contributed reagents/materials/analysis tools. NS, FK, and GP wrote the paper. JJ, MF, and OI critically revised the article for important intellectual content. All authors contributed to the article and approved the submitted version.

## Funding

This work was supported by grants from Fundação de Amparo a Pesquisa do Estado de São Paulo (FAPESP) and CAPES. MF and OI have CNPq fellowship.
